# Preliminary Quadriceps Muscle Contraction in the Early Rehabilitation of Hip and Knee Arthroplasty

**DOI:** 10.3390/jcm14197021

**Published:** 2025-10-03

**Authors:** Assen Aleksiev, Daniela Kovacheva-Predovska, Sasho Assiov

**Affiliations:** 1 Department of Physical Medicine and Rehabilitation, University Hospital “Aleksandrovska”, Medical University of Sofia, 1431 Sofia, Bulgaria; 2 Department of Physical Medicine and Rehabilitation, University Hospital “St. Anna”, Medical University of Sofia, 1750 Sofia, Bulgaria; didi.kovacheva@gmail.com; 3 Department of Orthopedics and Traumatology, University Hospital “St. Anna”, Medical University of Sofia, 1750 Sofia, Bulgaria; assiovmd@abv.bg

**Keywords:** hip arthroplasty, knee arthroplasty, early rehabilitation, preliminary quadriceps contraction

## Abstract

**Background**: Muscle latency is an often-overlooked factor contributing to increased implant wear and higher rates of hip and knee osteoarthritis. Latency reduces the protective role of muscles against external joint loads during movement initiation, leading to cumulative microtrauma. This study investigates whether preliminary quadriceps contraction can mitigate these adverse effects during early rehabilitation after arthroplasty. **Materials and methods**: The study was conducted in two university hospitals in Sofia, Bulgaria, including 46 patients (mean age 63.76 ± 9.49 years): 25 with hip arthroplasty and 21 with knee arthroplasty. Participants were randomly assigned to a control group (*n* = 25; 13 hip, 12 knee: standard postoperative advice) or an experimental group (*n* = 21; 12 hip, 9 knee: standard advice plus preliminary quadriceps contraction). Primary outcome: pain intensity (VAS). Secondary outcomes: range of motion (ROM, %), manual muscle testing (MMT, %), thigh circumference difference (cm), and success rate of preliminary quadriceps contraction (%). **Results**: Both groups improved after one month (*p* < 0.05), but the experimental group showed significantly greater improvement (*p* < 0.05). Higher success rates of preliminary quadriceps contraction correlated with greater improvements in all outcomes (*p* < 0.05). **Conclusions**: Preliminary quadriceps contraction enhances standard postoperative advice by reducing pain, improving mobility and muscle strength, and reducing hypotrophy during early rehabilitation after hip and knee arthroplasty. Patients should be encouraged to perform it consistently, even when pain subsides.

## 1. Introduction

The number of arthroplasty procedures has increased significantly worldwide, representing a substantial portion of healthcare expenditures [1]. Use of this technology has grown rapidly in recent years [2], with research in the field showing an average annual growth rate of 28% [3]. In Bulgaria, hip and knee arthroplasties have also risen sharply [4], with osteoarthritis as the leading indication [4,5].

Joint instability is one of the most frequent postoperative complications following hip and knee arthroplasty, contributing to increased implant wear [6]. Muscle latency is a critical factor in this process [7]. The monosynaptic reflex (M1) occurs at 50–60 ms, polysynaptic reflexes (M2) at 60–90 ms, triggered responses at 80–120 ms, proprioceptive/visual/vestibular responses at 120–150 ms, and voluntary reactions (M3) at 150–500 ms [8,9,10,11,12,13].

Hip and knee joint loading depends heavily on muscle-induced reaction forces [14,15,16]. During latency, immediate muscular protection is absent, allowing repetitive microtrauma to accumulate [8,9,10,11]. Latency increases with ageing, further raising the risk of injury [17,18].

The only effective strategy against the consequences of latency in hip and knee muscles is preliminary quadriceps contraction [11]. However, its role in early rehabilitation after arthroplasty has not yet been validated. While rehabilitation is crucial for ensuring surgical success, the optimal types and parameters of exercises remain uncertain [19,20,21,22]. Strengthening exercises play an important role [20,21], but alone they are insufficient to restore full muscle function and postural stability [19,23].

Preliminary quadriceps contraction induces reflex co-contraction of periarticular muscles, enhancing stability and strength [11]. Because these contractions occur frequently during daily activities, strengthening accumulates progressively without fatigue or adverse effects [11]. This not only reduces the impact of latency but also prevents microtraumas from escalating into macrotraumas [11]. Without this contraction, very high loads—several times a patient’s body weight, even during simple tasks such as sit-to-stand transfers [24,25,26]—impact the hip and knee joints unopposed during latency [11].

Previous studies have demonstrated the benefits of preliminary abdominal contraction in recurrent spinal pain [8,9], preliminary biceps brachii contraction in humeroscapular periarthritis [10], and preliminary quadriceps contraction in recurrent knee pain due to osteoarthritis [11]. Evidence regarding its application in hip and knee arthroplasty rehabilitation is lacking.

### Objective

To determine whether adding preliminary quadriceps contraction to standard postoperative advice improves early rehabilitation outcomes in hip and knee arthroplasty.

## 2. Materials and Methods

### 2.1. Study Setting

- Department of Physical Medicine and Rehabilitation, University Hospital “Aleksandrovska”, Sofia, Bulgaria;
- Department of Physical Medicine and Rehabilitation and Department of Orthopedics and Traumatology, University Hospital “St. Anna,” Sofia, Bulgaria.

### 2.2. Participants

**Figure 1:**
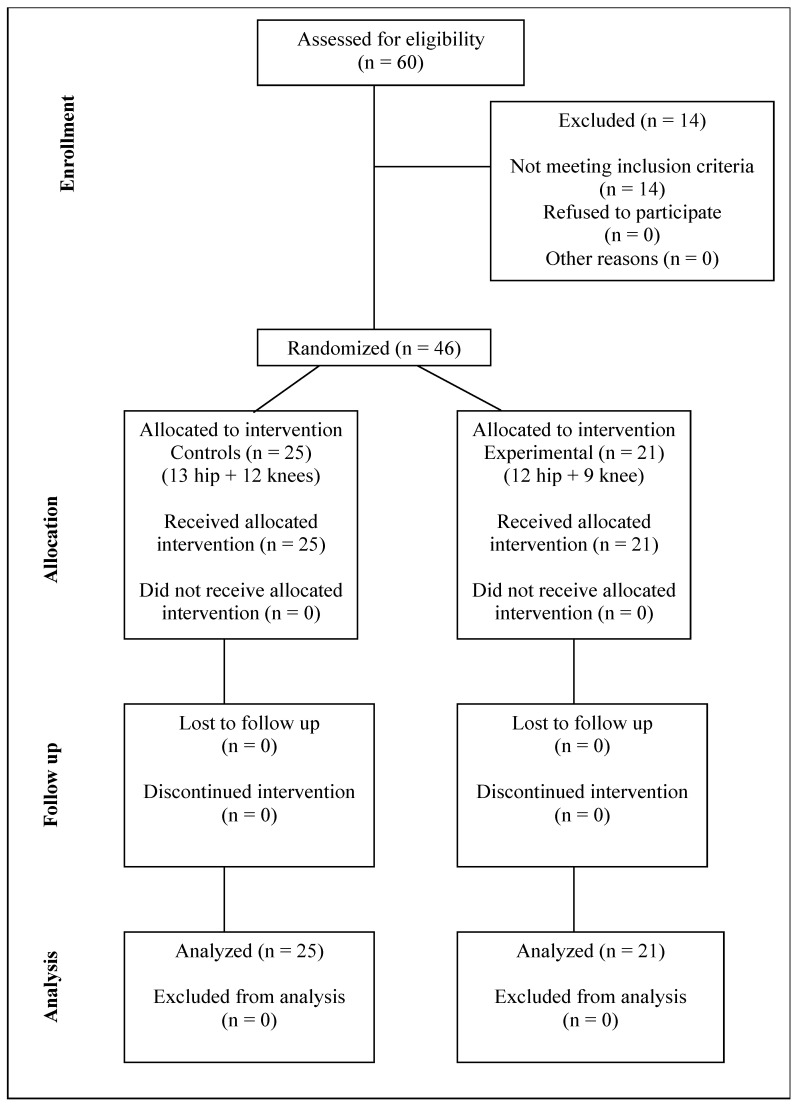
CONSORT diagram.

A total of 46 patients (mean age 63.76 ± 9.49 years; 25 hip arthroplasty, 21 knee arthroplasty) were enrolled (Figure 1).

Study Design: Randomized, parallel-assignment interventional study.

Randomization was computer-generated, with allocation concealed in sealed envelopes. Patients were blinded to group assignment. Outcome assessors (VAS, ROM, MMT, circumference) were also blinded.

### 2.3. Groups

- Control group (*n* = 25; 13 hip, 12 knee): standard postoperative advice.
- Experimental group (*n* = 21; 12 hip, 9 knee): standard advice plus preliminary quadriceps contraction.

### 2.4. Inclusion Criteria

- Age >18 years.
- Clinically and radiographically confirmed osteoarthrosis of the hip or knee, indicated for arthroplasty.
- Ability to ambulate independently or with an assistive device.

### 2.5. Exclusion Criteria

- Fractures.
- Revision arthroplasty.
- Severe comorbidities (pulmonary, cardiac, metabolic) limiting physical activity.
- Postoperative complications preventing discharge after day 7.
- Advanced osteoarthritis severely limiting rehabilitation participation.
- Aphasia, dementia, or psychiatric illness impairing participation.
- Blindness or illiteracy.

### 2.6. Interventions

Standard advice (all groups): general postoperative guidelines.

Patients were advised to mobilize early, verticalize, and walk with crutches or walkers, bearing up to 50% of body weight on the operated joint. They were instructed to avoid heavy physical activity, sudden or unexpected loads, repetitive/prolonged overload, and extended immobility. From postoperative day 1 to day 7, the rehabilitation program included frequent, short sessions of toe, ankle, and knee flexion–extension, ankle circumduction, hip abduction and flexion, and isometric leg muscle contractions in supine position. Patients were sat upright and verticalized to bedside standing on day 1. On day 2, they practiced short standing and walking with crutches or walkers in the room. Corridor walking was introduced on day 3, stair climbing on day 6, and activities of daily living after day 7. Specific precautions included weight-bearing on the healthy leg when sitting, with the operated leg extended forward and slightly bent. By day 9, patients practiced independent dressing/undressing (to the waist). From day 10, light exercises were introduced in prone position with higher intensity, and from day 15, patients practiced climbing stairs over multiple floors. Frequency, duration, and intensity of exercises were gradually increased to restore normal functioning by postoperative day 30.

### 2.7. Additional Advice (Experimental Group)

Patients were trained once in preliminary quadriceps contraction. Training lasted only seconds and was effective [11]. Tactile biofeedback was initially provided by placing a hand on the anterior thigh. Patients were instructed to contract the quadriceps before movements shifting the center of gravity (e.g., standing up, sitting down, bending, lifting). The maneuver required no extra time or equipment and was integrated into daily activities.

### 2.8. Outcome Measures

#### 2.8.1. Primary Outcome

**Pain intensity:** Pain intensity measured by the Visual Analog Scale (VAS) [27,28] (0 = no pain, 10 = “most severe pain imaginable”). Evaluations: day 1 post-op and day 30.

#### 2.8.2. Secondary Outcomes

Range of motion (ROM as percentage scores): Measured in degrees with a handheld goniometer [29,30,31,32,33]. For comparability, values were converted into percentages of normal ROM (0–100%). Average percentages were calculated for all directions and planes.

Muscle strength: Assessed by Manual Muscle Testing (MMT as percentage scores) [28,29,30,31,32], scored on a 0–5 scale (0 = no movement, 5 = full ROM against maximal resistance). For analysis, scores were converted to percentages of normal strength (0–100%). Average values were calculated for periarticular muscles of the hip and knee.

Muscle hypotrophy: Measured as the circumference difference (in cm) between affected and unaffected thighs [29,30,31,32,33].

Success rate of preliminary quadriceps contraction: Self-reported and defined as the percentage of movements before which patients performed the contraction [8,9,10,11]. For example, performing it before every second movement equaled 50%, every third = 33%, every fourth = 25%, etc. [8,9,10,11].

### 2.9. Sample Size and Power Analysis

1. ANOVA—Repeated Measures:
  - α = 0.05, Power = 0.95, Groups = 4, Measurements = 4, Corr = 0.5
  - Required *n* = 36, Achieved power = 0.951
2. Linear Multiple Regression:
  - α = 0.05, Power = 0.95, R^2^ deviation = 0.1578
  - Required *n* = 46, Achieved power = 0.950

The relatively small sample is acknowledged as a limitation.

### 2.10. Follow-Up

Outcomes were assessed at 30 days. The relatively short duration is acknowledged as a limitation.

### 2.11. Statistical Analysis

Data were analyzed using quantitative analysis of variance (ANOVA) to test statistical significance across the model and all interactions. Significant clusters were further analyzed with Bonferroni post hoc tests. Qualitative Pearson correlation analysis was used to examine interactions between individual parameters, and post hoc regression analysis was applied to calculate regression equations for significantly correlated variables.

## 3. Results

Baseline characteristics (age, weight, height, BMI, and outcome measures) did not differ significantly between groups (*p* > 0.05) (Table 1 and Table 2).

Both groups improved significantly after one month (*p* < 0.05), but the experimental group demonstrated greater improvements across all measures (*p* < 0.05) (Table 2).

Pain (VAS): Preoperative pain was comparable between control (5.28 ± 0.45 CI) and experimental (5.81 ± 0.60 CI) groups (*p* > 0.05). At one month, pain scores were lower in the experimental group (2.08 ± 0.21 CI) compared with controls (2.60 ± 0.23 CI) (*p* < 0.05).

Muscle strength (MMT as percentage scores): Preoperative strength was comparable between control (0.48 ± 0.03 CI) and experimental (0.46 ± 0.03 CI) groups (*p* > 0.05). At one month, strength was greater in the experimental groups (0.64 ± 0.04 CI) compared with controls (0.58 ± 0.02 CI) (*p* < 0.05).

Range of motion (ROM as percentage scores): Preoperative ROM was similar between control (0.25 ± 0.05 CI) and experimental (0.25 ± 0.07 CI) groups (*p* > 0.05). At one month, ROM was higher in the experimental group (0.44 ± 0.09 CI) compared with controls (0.38 ± 0.08 CI) (*p* < 0.05).

Thigh circumference difference: Preoperatively, the control (2.68 ± 0.25 CI) and experimental (2.76 ± 0.22 CI) groups did not differ (*p* > 0.05). At one month, the thigh circumference difference was smaller in the experimental group (0.71 ± 0.11 CI) compared with controls (1.08 ± 0.13 CI) (*p* < 0.05).

Significant correlations were found between outcomes (*p* < 0.05) (Table 3).

Higher success rates of preliminary quadriceps contraction were associated with reduced pain, increased strength and ROM, and reduced hypotrophy (Table 3). Regression analysis confirmed these relationships:

Pain (VAS) decreased with higher success rate:

VAS = 4.40 − (1.49 × Success rate) (R = 0.176; *p* = 0.023; F = 5.27).

Muscle strength (MMT as percentage scores) increased with a higher success rate:

MMT = 0.517 + (0.0842 × Success rate) (R = 0.182; *p* = 0.0188; F = 5.64).

Range of motion (ROM as percentage scores) increased with a higher success rate:

ROM = 0.307 + (0.0722 × Success rate) (R = 0.227; *p* = 0.003; F = 8.90).

Thigh circumference difference decreased with a higher success rate:

Circumference difference = 2.06 − (2.27 × Success rate) (R = 0.434; *p* = 0.0001; F = 38).

## 4. Discussion

Early rehabilitation after hip and knee arthroplasty is essential for optimizing recovery and reducing implant wear, though the ideal rehabilitation components remain debated [19,20,21]. Our study shows that preliminary quadriceps contraction significantly enhances standard postoperative advice, yielding additional benefits in pain reduction, mobility, muscle strength, and preservation of thigh circumference.

Quantitatively, all outcome measures—pain, muscle strength, range of motion, and thigh circumference—were significantly better in the experimental group compared with controls one month postoperatively, despite identical baseline values. The observed improvements in both groups reflect the effect of standard advice, yet the additional gains in the experimental group highlight the clinical utility of preliminary quadriceps contraction. Although an even more pronounced effect might have been observed if controls had received no advice, such a design would be ethically unacceptable. Despite the modest sample size, statistical significance was consistently achieved, further underscoring the robustness of the findings.

Qualitative analysis also confirmed these results. As expected, pain reduction correlated with improvements in muscle strength, range of motion, and reduced hypotrophy. However, the strong correlation and regression dependence of these outcomes on the success rate of quadriceps contraction emphasized the maneuver’s importance. Notably, the average success rate was only 43% ± 23%, below the 50% threshold expected by chance. Achieving 100% would require continuous vigilance-contracting the quadriceps before every movement shifting the body’s center of gravity. In practice, many patients tended to abandon the maneuver once pain subsided, thereby losing its preventive and prophylactic effect. This finding suggests that patients must be encouraged to maintain the practice even in the absence of pain, as its benefits extend beyond symptom control.

Interestingly, the intervention’s effects did not differ between hip and knee arthroplasty patients, despite biomechanical differences between these joints. This likely reflects the central role of the quadriceps muscle in both hip and knee function, particularly in response to pathology, surgery, and recovery.

Evidence consistently suggests that preliminary contraction reduces microtrauma accumulation due to muscle latency. Our findings support incorporating preliminary quadriceps contraction into postoperative rehabilitation guidelines, adapted to individual patient needs. They also align with our earlier work on preliminary abdominal muscle contraction in recurrent spine pain [8,9], biceps brachii contraction in humeroscapular periarthritis [10], and quadriceps contraction in recurrent knee pain due to osteoarthritis [11]. While previous studies have emphasized the role of strengthening and structured rehabilitation after arthroplasty [19,20,21], our results highlight the unique added value of integrating preliminary quadriceps contraction into these protocols. To our knowledge, no comparable studies have evaluated this maneuver specifically in hip and knee arthroplasty, making our study a novel contribution.

### Study Limitations

- Small sample for drawing strong generalizable conclusions;
- Short follow-up (30 days);
- Relatively subjective outcome measures;
- Self-reported adherence;
- Lack of evaluation of neuromuscular deficits such as arthrogenic muscle inhibition (AMI) [34,35,36], hyperexcitability of flexion withdrawal reflexes [7], altered hamstring-to-quadriceps ratios [37,38], impaired force control, voluntary activation deficits, changes in cortical and spinal excitability, reflex modulation, torque variability [37,38], or electromechanical delay [37,38]. Further studies are needed to assess their role, including larger cohorts, longer follow-ups, objective monitoring and adherence verification.

## 5. Conclusions

Preliminary quadriceps contraction enhances standard postoperative rehabilitation after hip and knee arthroplasty by reducing pain and improving joint mobility, strength, and muscle preservation. Patients should be encouraged to practice the maneuver consistently, even when asymptomatic, to maximize its long-term protective and functional benefits.

## Figures and Tables

**Table 1:** Baseline outcomes.

Groups	Control with Hip Arthroplasty	Control with Knee Arthroplasty	Experimental with Hip Arthroplasty	Experimental with Knee Arthroplasty
Number of patients	13	12	12	9
Age (years)	61.92 ± 5.18(CI)	68.58 ± 4.11(CI)	68.17 ± 6.34(CI)	67.44 ± 5.57(CI)
Weight (kg)	81.0 ± 12.80(CI)	85.08 ± 15.55(CI)	85.33 ± 11.71(CI)	83.44 ± 13.11(CI)
Height (cm)	167.31 ± 6.05(CI)	162.67 ± 7.32(CI)	168.83 ± 5.74(CI)	159.89 ± 7.26(CI)
Body mass index	22.29 ± 4.19(CI)	26.71 ± 5.30(CI)	20.76 ± 4.27(CI)	26.69 ± 4.27(CI)

(CI)—confidence interval.

**Table 2:** Primary and secondary outcomes before and after operation.

Groups	Control	Control	Experimental	Experimental
Number of patients	25	25	21	21
Follow-up	Before surgery	After 1 month	Before surgery	After 1 month
Visual analog scale	5.28 ± 0.45(CI)	2.6 ± 0.23(CI)	5.81 ± 0.60(CI)	2.08 ± 0.21(CI)
Manual muscle testing	0.48 ± 0.03(CI)	0.58 ± 0.02(CI)	0.46 ± 0.03(CI)	0.64 ± 0.04(CI)
Range of motion	0.25 ± 0.05(CI)	0.38 ± 0.08(CI)	0.25 ± 0.07(CI)	0.44 ± 0.09(CI)
Thigh circumference difference	2.68 ± 0.25(CI)	1.08 ± 0.13(CI)	2.76 ± 0.22(CI)	0.71 ± 0.11(CI)

(CI)—confidence interval.

**Table 3:** Pearson correlation between the primary and secondary outcomes.

	MMT	ROM	Success Rate	Difference (cm)
VAS Correlation coefficient *p*-value	Correlation −0.553 <0.001	Correlation −0.255059 <0.001	Correlation −0.1764 <0.03	Correlation +0.22459 <0.005
MMT Correlation coefficient *p*-value		Correlation +0.3225093 <0.001	Correlation +0.1823 <0.02	Correlation –0.515400 <0.001
ROM Correlation coefficient *p*-value			Correlation +0.49904 <0.001	Correlation –0.5379053 <0.001
Success rate Correlation coefficient *p*-value				Correlation −0.4335101521 <0.001

The pairs of variables with positive correlation coefficients and *p*-values below 0.05 tend to increase together. For the pairs with negative correlation coefficients and *p*-values below 0.05, one variable tends to decrease while the other increases. “VAS”—visual analog scale; “MMT”—manual muscle testing as percentage scores; “ROM”—range of motion as percentage scores; “Success rate”—preliminary quadriceps muscle contraction success rate; “Difference (cm)”—circumference difference (between affected and unaffected thigh) in centimeters.

## Data Availability

The raw data supporting the conclusions of this article will be made available by the authors on request.

## Abbreviations

The following abbreviations are used in this manuscript:

VAS	Visual analog scale
MMT	Manual muscle testing
ROM	Range of motion
Pre-Contr	preliminary contraction
BMI	body mass index
AMI	arthrogenic muscle inhibition
FWRs	hyperexcitability of flexion withdrawal reflexes
CI	confidence interval
